# Heterogeneous microcommunities and ecosystem multifunctionality in seminatural grasslands under three management modes

**DOI:** 10.1002/ece3.2604

**Published:** 2016-11-27

**Authors:** Jingpeng Li, Zhirong Zheng, Hongtao Xie, Nianxi Zhao, Yubao Gao

**Affiliations:** ^1^ College of Life ScienceNankai UniversityTianjinChina; ^2^ State Environmental Protection Key Laboratory of Regional Eco‐Process and Function AssessmentChinese Research Academy of Environmental SciencesBeijingChina; ^3^ Research Institutes of Subtropical ForestryChinese Academy of ForestryFuyangZhejiang 311400China

**Keywords:** biodiversity, grassland, microcommunity, multifunctionality, species richness, TWINSPAN

## Abstract

Increasing attention has been paid to the relationship between biodiversity and ecosystem functioning (BEF) because of the rapid increase in species loss. However, over the past 20 years, most BEF studies only focused on the effect of species diversity on one or a few ecosystem functions, and only a few studies focused on ecosystem multifunctionality (i.e., the simultaneous provision of several ecosystem functions). Grassland ecosystems have important economic, environmental, and esthetic value; thus, this study focused on the heterogeneous microcommunities in grasslands under three management modes. The multifunctionality index (*M‐index*) was assessed at community and microcommunity scales, and the relationship between species diversity and multifunctionality was investigated. The communities were found to be respectively composed of one, three, and six microcommunities in grazing, clipping, and enclosure management, based on a two‐way indicator species analysis (TWINSPAN) and detrended correspondence analysis (DCA) for community structure. Biodiversity and soil indicators showed an apparent degradation of the grazing community, which had the worst *M‐index*. Clipping and enclosure communities showed no significant difference in biodiversity indices, soil variables, and *M‐index*; however, these indices were clearly different among microcommunities. Therefore, the microcommunity scale may be suitable to investigate the relationship between vegetation and multifunctionality in seminatural grassland ecosystems. Dominant species richness had more explanatory power for ecosystem multifunctionality than subdominant species richness, rare species richness, and the number of all species. Therefore, it is important to distinguish the role and rank of different species in the species richness–multifunctionality model; otherwise, the model might include redundant and unclear information. Communities with more codominant species whose distribution is also even might have better multifunctionality.

## 1 Introduction

Global change is altering biodiversity worldwide at an unprecedented speed, resulting in unpredictable consequences for ecosystem functions (Cardinale et al., 2012; Grime, 1997; MacDougall, McCann, Gellner, & Turkington, 2013; Valencia et al., 2015). In particular, with the dramatic decline in biodiversity, researchers have increasingly focused on the relationships among the changed biodiversity, community structure, and ecosystem functioning (BEF) (Diaz & Cabido, 2001; Diaz et al., 2007; Gamfeldt, Hillebrand, & Jonsson, 2008; Grime, 1997; Hector & Bagchi, 2007; Tilman et al., 1997). Over the past 20 years, most studies only focused the effect of species diversity on one or a few ecosystem functions (Hector et al., 1999; Simova, Li, & Storch, 2013; Valencia et al., 2015; Waide et al., 1999), and few evaluated multiple ecosystem functions. However, ecosystems are primarily valued because they provide multiple functions and services simultaneously (i.e., multifunctionality) (Gamfeldt et al., 2008; Hector & Bagchi, 2007; Sanderson et al., 2004; Soliveres et al., 2014). Therefore, assessing how human management activity may impact multifunctionality is crucial to understand the ecological consequences of human management on seminatural grasslands (Byrnes et al., 2014; Maestre, Castillo‐Monroy, Bowker, & Ochoa‐Hueso, 2012; Soliveres et al., 2014; Valencia et al., 2015; Wagg, Bender, Widmer, & Heijden, 2014).

Hector and Bagchi (2007) defined multiple ecosystem services or processes as ecosystem multifunctionality and quantified the effects of species diversity on multiple combined ecological processes for the first time. Gamfeldt et al. (2008) and Zavaleta, Pasari, Hulvey, and Tilman (2010) also defined and quantified ecosystem multifunctionality separately. They considered that multifunctionality was the ability to maintain multiple ecological functions or services simultaneously and that sustaining multiple ecosystem functions in grassland communities requires higher biodiversity. Maestre, Quero, et al. (2012) used 14 soil variables (reflecting ecological processes of the C, N, and P cycles) to synthetically evaluate ecosystem multifunctionality in global dryland, and the evaluation method and indicators in this study have been widely adopted in recent years by studies that assess ecosystem multifunctionality (Byrnes et al., 2014; Soliveres et al., 2014; Valencia et al., 2015; Wagg et al., 2014).

In China, the grassland area is 3.55 × 10^8^ hm^2^, accounting for approximately 41.7% of the territorial area and having important economic, environmental, and esthetic value (Yan et al., 2014); however, human activities have seriously affected the plant distribution, community structure, and ecosystem functions of the natural grassland communities (Xu, Gao, & Wang, 2014). Therefore, investigating the change in the structure and ecosystem function of grassland communities is important for the sustainable management and development of grassland. Grazing, clipping, and enclosure are three main grassland management modes, and they result in different succession states for grassland communities and generate different interspecific and intraspecific relationships, causing heterogeneous community structure. Heterogeneous microcommunities (or dependent communities) are notably different in species composition and appearance from the background community they belong to and are composed of different types of plants. The formation and existence of microcommunities are largely dependent on the gramineous background community, and the microcommunities are a part of the horizontal structural differentiation of the overall community (Chen & Li, 2011; Song, 2001). Because of the differences in species composition and microhabitat conditions among different microcommunities, the change rules in multifunctionality would be more distinct at the microcommunity scale. Therefore, this study aimed to (1) analyze the heterogeneous microcommunity composition in a grassland ecosystem under three management modes according to community classification and ordination, (2) compare the change rules of multifunctionality at the microcommunity and community levels to determine the appropriate scale for researching multifunctionality, and (3) investigate the relationship between species diversity and multifunctionality to determine the relative importance of different species (dominant species, subdominant species, and rare species) for changes in multifunctionality.

## 2 Materials and Methods

### 2.1 Study site

The experiment was conducted in Huihe National Nature Reserve in Hulunbeier Grassland, Inner Mongolia (118°48–119°45′E, 48°10–48°57′N), which has a total area of 3468 km^2^. The regional climate is a temperate continental monsoon climate. The annual average temperature is −2.4 to 2.2°C. The frost‐free period is 100–120 days, and the annual average precipitation in 2008–2014 was 375.03 mm, of which 70% was concentrated between June and August. Zonal soil types are chernozem and chestnut (Li, Zheng, Ye, Xia, & Feng, 2014).

### 2.2 Experiment design and sampling

**Figure 1:**
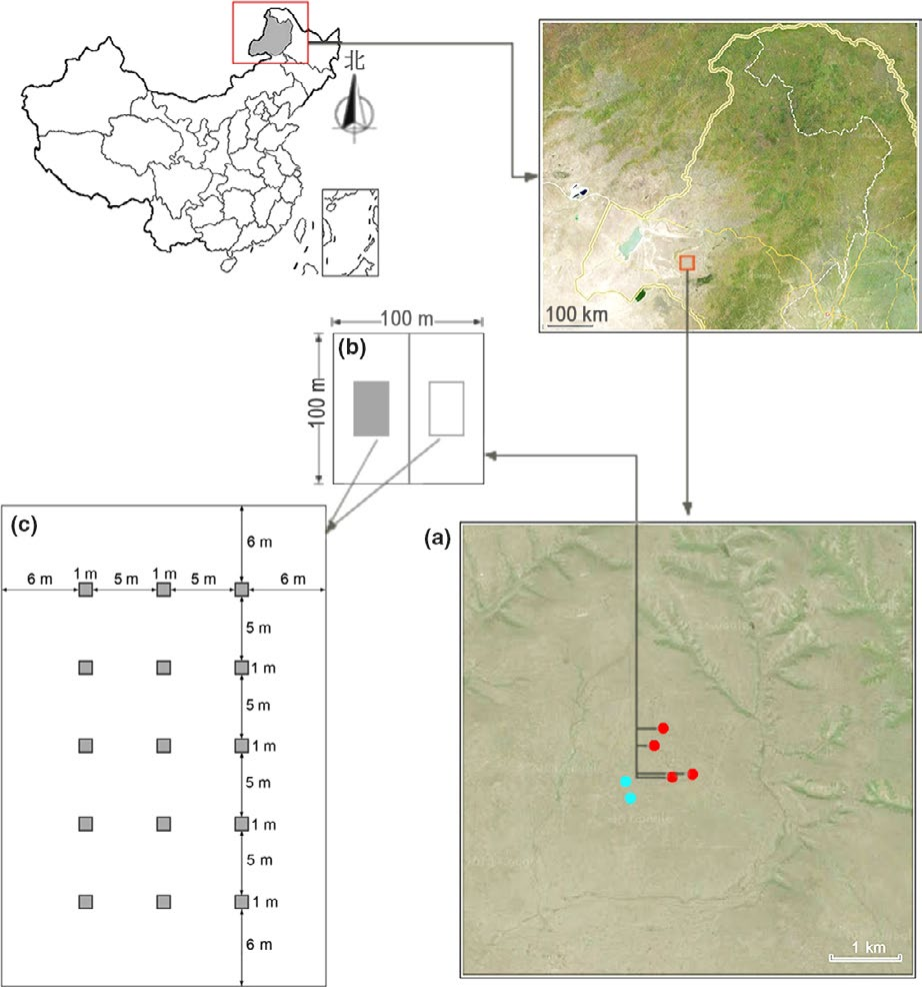
Study area and sites setting in Hui He National Nature Reserve, Inner Mongolia. Red circles represent 4 study sites in fenced‐off area (a); each site was divided two parts, conducting clipping treatment and enclosure treatment, respectively. (b). In 2014, one plot of 25 m × 37 m was set in the center of each treatment, and 15 quadrats of 1 m × 1 m were set at equal interval in each plot. (c) A total of 60 clipping quadrats and 60 enclosure quadrats were recorded in four study sites. Blue circles represent two grazing sites within the public stock road outside fenced‐off area

In June 2008, we set four study sites of 1 ha per site in a local herder's pasture with uniform and smooth terrain that was used as a free‐grazing pasture before 2008 (Figure 1a). According to the most common grassland management modes in the local region, each site was randomly divided into two parts (each part had a size of 0.5 ha) to conduct clipping and enclosure management, respectively (Figure 1b). In the enclosure parts, no grazing or human disturbance occurred in the entire experiment period, whereas in the clipping parts, grass was cut once every year around August 20 (local grass‐mowing time). In August 2014 at the peak of biomass, we established one plot of 25 m × 37 m in the center of each clipping and enclosure part, respectively. In each plot, we set 15 quadrats of 1 m × 1 m per quadrat at equal intervals (Figure 1c); thus, 60 clipping quadrats and 60 enclosure quadrats were recorded in total in four study sites.

Because our study sites were located in a herder's pasture (rented from a local herder) where grazing was forbidden according to local pasture utilization status, we were not able to conduct grazing treatment in the above‐mentioned four study sites. However, as a reference, two grazing sites (50 m × 100 m per site) were set within the public grazing path (the road that livestock crossed to pasture) outside the pasture where the above‐mentioned four study sites were set, and the grazing intensity was found to be approximately 62.3 standard sheep unit/km^2^ (equivalent to the local actual free‐grazing intensity). As the grazing community showed highly a homogenized structure, we set 25 quadrats of 1 m × 1 m along the grazing path direction. In addition, the species–area curves of communities under three management modes (Appendix S5) showed that no new species emerged when the number of sampling quadrats reached 19; therefore, we only selected 20 grazing quadrats in the subsequent experiments. Then, we recorded the presence of all plant species and determined their density, coverage, mean height, and relative frequency (RFi) in each quadrat under the three management modes.

### 2.3 Soil sampling and analyses

**Figure 2:**
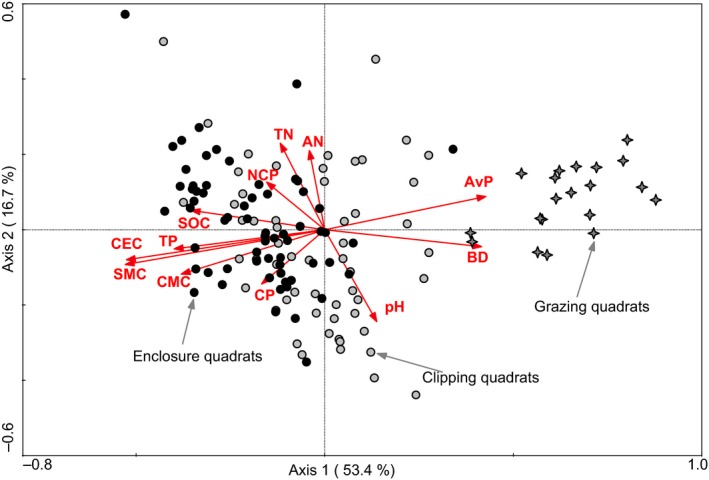
Redundance analysis showing the effect of soil variables on the distribution patterns of plant quadrats

Soil cores (0–15 cm depth) were sampled using the quincunx sampling method to obtain a mixed sample for each quadrat. We thus collected 60 soil samples for both clipping and enclosure communities, and 20 samples for the grazing community. Maestre, Castillo‐Monroy, et al. (2012), Maestre, Quero, et al. (2012) and Valencia et al. (2015) respectively selected nine to 14 soil variables to evaluate ecosystem multifunctionality. Here, we selected the following 12 variables to assess multifunctionality: pH, total nitrogen (TN), available nitrogen (AN), soil organic carbon (SOC), total phosphorus (TP), available phosphorus (AvP), soil moisture content (SMC), bulk density (BD), capillary moisture capacity (CMC), cation exchange capacity (CEC), capillary porosity (CP), and noncapillary porosity (NCP). These variables constitute a good proxy for processes such as nutrient cycling, biological productivity, and buildup of nutrient pools, which are important determinants of ecosystem functioning (e.g., water and soil conservation, soil respiration, and carrying of flora and fauna) in dry lands (Valencia et al., 2015). Most of these processes are also considered to support ecosystem services, as other types of ecosystem services depend on them (Isbell et al., 2011). Redundance analysis (RDA) (Figure 2) also confirmed that the selected 12 soil variables had a significant influence on the plant distribution (*p *=* *.0020, Monte Carlo test). The first two environmental axes explained 70.1% of the total information, and the first axis was mainly determined by factors such as CEC, SMC, CMC, BD, and AvP, which explained as much as 53.4% of the information content.

### 2.4 Data processing

#### 2.4.1 Species importance values and biodiversity

The species importance values (IV) under different management modes were calculated as follows:

(1) $${\text{IV}}_{i}={\text{RD}}_{i}+{\text{RC}}_{i}+{\text{RF}}_{i}$$

where RD_*i*_, RC_*i*_, and RF_*i*_ represent the relative species density, relative coverage, and relative frequency, respectively.

The biodiversity index includes the Simpson index, Margalef index, and Evenness index as described below (Song, 2001):

(2) $$\text{Simpson index}:\;D=1-\sum P_{i}^{2}$$

(3) Margalef index:Ma=(S−1)/lnN

(4) $$\text{Evenness(Pielou) index}:\;J_{\mathrm{sw}}=H^{\prime }/\text{ln}S$$

where *i* is the *i*th species, *S* is the number of species, *N* is the total number of individuals of a species occurring within the quadrats, and *P*_*i*_ is the relative density of the ith species.

#### 2.4.2 TWINSPAN clustering and DCA ordination

According to the quadrat data from the three management modes, a quadrat–species matrix based on the coverage of each species was first established and then classified into four levels using two‐way indicator species analysis (TWINSPAN) in PC‐ORD 6.0, and finally, the classification results for the quadrats and species were obtained. Furthermore, a quadrat–species matrix based on coverage was sorted and mapped using detrended correspondence analysis (DCA) in CANOCO 4.5. Combining the results of TWINSPAN clustering (Appendix S3) and DCA ordination (Appendix S2), we obtained the main microcommunities under the three management modes (Appendix S1).

### 2.5 Multifunctionality index

Multifunctionality was estimated from all of the soil variables measured using the *M‐index* of Maestre, Castillo‐Monroy, et al. (2012). To obtain the *M‐index* for each community, *Z*‐scores were first calculated for each of the 12 soil variables estimated at each quadrat surveyed. Raw data were normalized before calculations; a square root transformation normalized most of the variables evaluated. The *Z*‐scores of the 12 soil variables were averaged to obtain the *M‐index* in each quadrat. This index provides a straightforward and easily interpretable measure of the ability of different communities to sustain multiple ecosystem functions simultaneously (Byrnes et al., 2014). It is also statistically robust (Maestre, Castillo‐Monroy, et al., 2012) and is being increasingly used when assessing multifunctionality (Quero et al., 2013; Bradford et al., 2014; Pendleton et al., 2014; Wagg et al., 2014). Moreover, the relatively large number of variables employed to calculate the *M‐index* makes it relatively robust to outliers or atypical values.

### 2.6 Multifunctionality index among microcommunities

Based on TWINSPAN and DCA results, we divided communities under three management modes into 10 microcommunities (Appendix S1) and selected the most typical quadrats from the 10 microcommunities mentioned above to compare *M‐index*. The whole grazing community was treated as one microcommunity (marked as G1) containing 20 quadrats; the enclosure community included three microcommunities (marked as E1, E2, and E3), each type containing 10 quadrats, and the clipping community included six microcommunities (marked as C1, C2, C3, C4, C5, and C6), each type containing five quadrats. First, we square root‐transformed the 12 soil variables and biodiversity indices to meet the required assumption of normality of the dependent variables in further statistical treatments. We used a mixed‐model approach to compare the differences for each index between the clipping community and enclosure community, among which soil variables, biodiversity indices, and *M‐index* were regarded as independent variables, management modes were the fixed factor, and the four study sites were random variables. Then, to compare the indices between the grazing community and clipping community and between the grazing community and enclosure community, we conducted independent samples t‐tests. Because of the high homogeneity of the grazing community structure, almost no heterogeneous microcommunity appeared; therefore, we only considered the microcommunity composition under clipping and enclosure management and applied one‐way ANOVA and LSD tests to compare the biodiversity indices and *M‐index* among the respective microcommunities under the clipping and enclosure management modes. We also used multiple independent sample nonparametric tests (Kruskal–Wallis *H*‐tests) to calculate the mean ranks of the soil variables, diversity indices, and *M‐index* of all microcommunities under the three management modes.

### 2.7 Species abundance class

To facilitate the analysis and interpretation, each species was classified as a dominant species (relative abundance ≥10%), subdominant species (1% ≤relative abundance <10%), or rare species (relative abundance <1%) based on its relative abundance (density or coverage) in the whole community under the three management modes (Clark & Tilman, 2008). Using the *M‐index* as a response variable, four species richness indexes as fixed factors, and microcommunity types as a random factor, we then built a multilevel mixed model to analyze the effect of the number of species with different statuses on ecosystem function and to examine the effect of the richness (number) of dominant species, subdominant species, and rare species on *M‐index*. A simple correlation analysis was adopted to investigate the relationships between biodiversity indices and *M‐index*. Statistical analyses were performed using Statistica 8 and SPSS 17.

## 3 Results

### 3.1 Soil variables and plant diversity in communities under three management modes

Biodiversity indices and most soil variables all had significantly worse values in the grazing community than in the clipping and enclosure communities, except for AvP, pH, and TN (Appendix S4); therefore, the grazing community had the lowest *M‐index* (Appendix S4). Soil variables and plant biodiversity in the grazing community showed serious degradation, but these indicators and ecosystem *M‐index* were not significantly different between the clipping and enclosure communities, and the data from the different sites also did not show significant aggregation (Table 1).

**Table 1:** Results of mixed‐model analyses for biodiversity indices, soil variables, and *M‐index* in clipping and enclosure communities

	Simpson index		Marglef index		Evenness index		*M‐index*	
	Estimate	*p*	Estimate	*p*	Estimate	*p*	Estimate	*p*
Management mode	−0.23	.21	−0.19	.24	−0.27	.14	0.06	.71
Site	0.05	.47	0.36	.26	0.07	.42	0.29	.26

	TN		AN		CEC		NCP	
	Estimate	*p*	Estimate	*p*	Estimate	*p*	Estimate	*p*
Management mode	0.49	.001**	0.21	.16	−0.39	.01*	−0.25	.18
Site	0.42	.24	0.48	.24	0.41	.25	0.05	.47

	TP		AvP		SMC		SOC	
	Estimate	*p*	Estimate	*p*	Estimate	*p*	Estimate	*p*
Management mode	−0.54	.001**	−0.54	<.001**	0.75	<.001**	−0.09	.56
Site	0.31	.26	0.17	.28	0.00	.87	0.47	.24

	CMC		CP		pH		BD	
	Estimate	*p*	Estimate	*p*	Estimate	*p*	Estimate	*p*
Management mode	0.31	.08	0.33	.067	0.06	.72	−0.11	.55
Site	0.13	.32	0.11	.34	0.28	.27	0.06	.42

Biodiversity indices and soil variables were treated as dependent variables. Management mode (clipping and enclosure) was treated as a fixed–effect factor. “Site” (*n* = 4) was treated as random factor to address the nonindependence of quadrats in the same sites. *p <* *.05; **p <* *.001.

### 3.2 Heterogeneous community composition and microcommunity types

**Figure 3:**
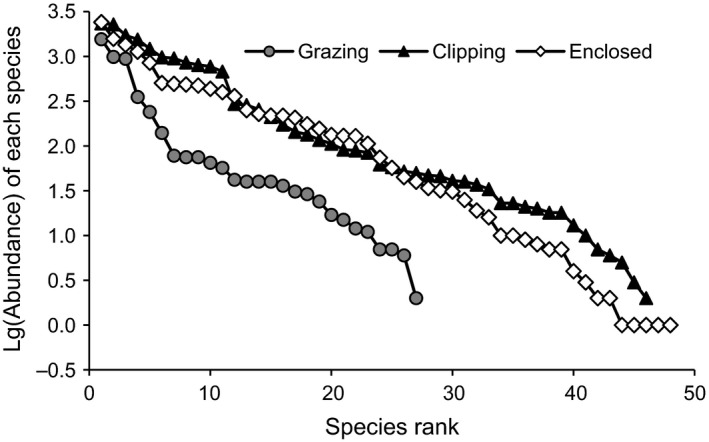
Species rank abundance curves of the communities under three management modes

No significant differences were observed in biodiversity indices between clipping and enclosure communities at the community level, but the species composition and community structure were not identical. The species rank abundance curve showed that the species number (5) of the first superiority rank in the enclosure community was obviously lower than that in the clipping community (10) (Figure 3). In the enclosure community, the dominant species, *Leymus chinensis* and *Artemisia capillaries,* were in the absolute dominant position and had an *IV* significantly higher than that of the other dominant species; additionally, species that had only one individual also occupied a certain proportion (Figure 3, Table 2). In the clipping community, the species *IV* showed little difference among different dominant species (Table 2), indicating that the distribution of dominant species in the clipping community was more uniform than that in the enclosure community and that the clipping community had more microcommunities dominated by different dominant species.

**Table 2:** Quantity characteristics of main common species in three communities

Community	Species	Abundance	Frequency	Coverage	Relative abundance	Relative frequency	Relative coverage	IV
Grazing	*Cleistogenes squarrosa*	1558	25	622	31.58	9.96	39.32	80.86
	*Potentilla acaulis*	943	22	316	19.12	8.76	19.97	47.86
	*Carex duriuscula*	990	23	165	20.07	9.16	10.43	39.66
	*Leymus chinensis*	353	24	43	7.16	9.56	2.72	19.44
	*Stipa grandis*	140	17	33	2.84	6.77	2.09	11.70
	*Serratula centauroides*	78	18	39	1.58	7.17	2.47	11.22
	*Artemisia frigida*	75	12	60	1.52	4.78	3.79	10.09
	*Potentilla chinensis*	57	16	35	1.16	6.37	2.21	9.74
	*Eurotia ceratoides*	240	4	43	4.87	1.59	2.72	9.18
	*Plantago asiatica*	65	10	44	1.32	3.98	2.78	8.08
Clipping	*Cleistogenes squarrosa*	1217	58	630	7.26	7.39	15.22	29.86
	*Leymus chinensis*	2333	56	236	13.91	7.13	5.70	26.75
	*Artemisia capillaries*	1550	53	402	9.24	6.75	9.71	25.71
	*Poa sphondylodes*	2282	49	202	13.61	6.24	4.88	24.73
	*Carex duriuscula*	1722	57	144	10.27	7.26	3.48	21.01
	*Serratula centauroides*	679	56	358	4.05	7.13	8.65	19.83
	*Potentilla acaulis*	953	39	301	5.68	4.97	7.27	17.92
	*Caragana microphylla*	979	28	335	5.84	3.57	8.09	17.50
	*Bupleurum tenue*	856	49	233	5.10	6.24	5.63	16.98
	*Artemisia frigida*	804	23	293	4.79	2.93	7.08	14.80
Enclosure	*Leymus chinensis*	2412	60	616	18.73	8.20	13.00	39.93
	*Artemisia capillaries*	1581	50	728	12.28	6.83	15.37	34.47
	*Cleistogenes squarrosa*	1352	54	490	10.50	7.38	10.34	28.22
	*Caragana microphylla*	486	33	487	3.77	4.51	10.28	18.56
	*Serratula centauroides*	506	41	374	3.93	5.60	7.89	17.42
	*Carex duriuscula*	1131	47	87	8.78	6.42	1.84	17.04
	*Potentilla bifurca*	495	47	239	3.84	6.42	5.04	15.31
	*Artemisia frigida*	851	19	209	6.61	2.60	4.41	13.61
	*Bupleurum tenue*	399	37	187	3.10	5.05	3.95	12.10
	*Cymbaria dahurica*	361	26	90	2.80	3.55	1.90	8.25

The DCA ordination and TWINSPAN clustering also confirmed that the clipping community had more heterogeneous microcommunities and that the enclosure community showed fusion of patches, formed from two main microcommunities dominated by *Leymus chinensis* and *Artemisia capillaries* (Appendix S2, S3). Based on the DCA and TWINSPAN results, we divided the three communities into 10 microcommunities (Appendix S1).

### 3.3 Soil variables, biodiversity, and multifunctionality based on microcommunities

**Figure 4:**
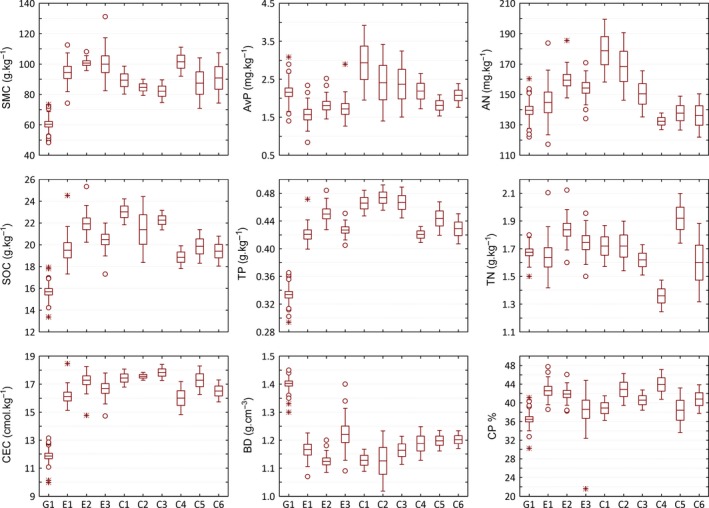
The differences in main soil variables among microcommunities. The horizontal line in each box is the mean. Boxes indicate the mean ± *SE*; I, mean ± *SD*; ○, outliers; *, extremes

There were no significant differences in soil variables, biodiversity indices, and *M‐index* between clipping and enclosure management at the community scale, but significant differences were observed at the microcommunity scale (Figure 4, Tables 3 and 4). Among the three microcommunities in the enclosure community, E2 was dominated by codominant species, whereas E1 and E3 were dominated by monodominant species, that is, *Leymus chinensis* and *Artemisia capillaries*, respectively. Dominant species and subdominant species richness and evenness were higher in E2 than those in E1 and E3 (Tables 3 and 4), whereas the values of major soil variables including BD, AN, TN, TP, SOC, and CEC in E2 were also obviously better than those in E1 and E3; therefore, E2 had a better *M‐index* than E1 and E3 (Tables 3 and 4). In the clipping community, the values of the soil variables CEC, TN, AN, TP, and SOC were all worst in C4; therefore, its M‐index was the lowest. Because C1 had the highest content of AvP, AN, and SOC, it also had the highest M‐index. Additionally, the number of dominant species in the clipping community in C1 was higher than that in C4, which had higher rare species richness (Table 3).

**Table 3:** The mean rank of biodiversity indices among microcommunities by Kruskal–Wallis *H*–test

Microcommunity	Mean rank of Kruskal–Wallis *H*–test							
	Dominant species richness	Subdominant species richness	Rare species richness	All species richness	Evenness index	Margalef index	Simpson index	*M–index*
G1	20.38	18.30	40.45	17.60	17.18	14.25	15.33	11.45
E1	26.00	54.55	30.40	41.25	43.25	43.15	42.80	38.60
E2	36.40	63.95	24.55	49.00	58.65	57.25	57.05	67.40
E3	34.15	44.80	25.20	33.80	38.95	35.85	35.85	43.90
C1	69.50	18.40	31.60	30.70	58.10	44.80	51.00	65.00
C2	59.00	30.70	52.30	44.00	25.90	32.60	28.30	54.40
C3	69.50	40.00	48.20	54.80	65.30	63.70	66.40	56.80
C4	43.40	49.00	59.60	56.00	55.10	59.80	61.20	35.20
C5	66.00	52.70	73.60	75.50	22.00	41.40	33.50	49.80
C6	66.00	57.40	60.60	68.50	71.20	76.20	74.90	41.20
χ^2^ (*n* = 80)	61.038	46.533	25.921	45.740	50.848	52.238	53.036	55.809
*p* (Asymp. Sig.)	<.001	<.001	.002	<.001	<.001	<.001	<.001	<.001

**Table 4:** The difference of biodiversity indices and *M–indexes* among different microcommunities in enclosure and clipping communities

Management modes	Microcoenosium (*n*)	Biodiversity index			*M–index*
		Simposon index	Margalef index	Evenness index	
Enclosure	E1(10)	0.82 ab	2.04 b	0.82 ab	−0.26 b
	E2(10)	0.86 a	2.39 a	0.87 a	0.35 a
	E3(10)	0.79 b	1.99 b	0.80 b	−0.09 b
Clipping	C1(5)	0.84 ab	1.87 b	0.87 a	0.28 a
	C2(5)	0.68 b	2.10 b	0.68 b	0.17 ab
	C3(5)	0.88 b	2.43 a	0.89 a	0.13 ab
	C4(5)	0.86 b	2.45 a	0.85 a	−0.30 c
	C5(5)	0.76 ab	2.53 a	0.68 b	−0.05 abc
	C6(5)	0.90 b	2.60 a	0.92 a	−0.23 bc

Same lowercase letters indicate the nonsignificant difference among microcommunities in the enclosure community or clipping community by LSD test.

### 3.4 Relationship between species diversity and ecosystem multifunctionality

**Figure 5:**
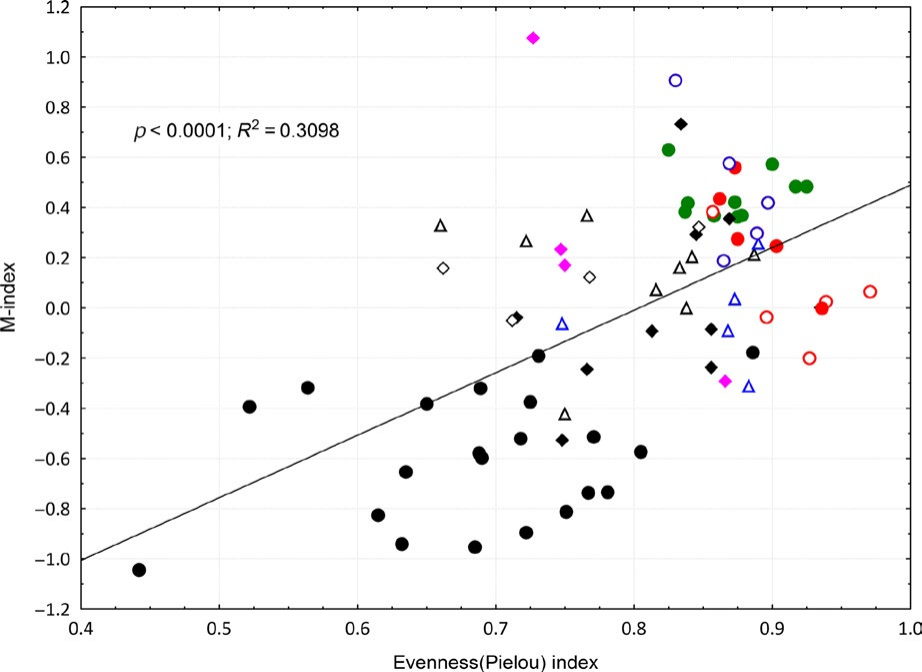
The relationship between evenness indexes and *M‐indexes* (multifunctionality index) in all quadrats. Symbols with different colors represent the quadrats of different microcommunities

*M‐index* was positively correlated with all diversity indices, suggesting that increasing diversity can enhance ecosystem multifunctionality. *M‐index* had a higher correlation with evenness (*R* ^2^ = .3098, *p *<* *.0001) than with the Simpson index (*R* ^2^ = .0998, *p *=* *.0043) and Shannon–Wiener index (*R* ^2^ = .1611, *p *=* *.0002) (Figure 5), showing that an even distribution of species may be more important than increased species number for improvement of multifunctionality. The rare species richness had no significant effect on *M‐index* (*p *=* *.401), and subdominant species richness had a weak effect (*R* ^2^ = .141, *p *=* *.014), whereas the influence of dominant species richness was most significant (*R* ^2^ = .218, *p *<* *.001). Dominant species richness also had a greater explanatory power for *M‐index* than the number of all species (including dominant, subdominant, and rare species) (Table 5). Therefore, communities with more dominant species that are evenly distributed might have better multifunctionality.

**Table 5:** Dependence of *M–index* on species diversity by multilevel mixed–model analysis (*n* = 80)

Response variable	Explanatory variable	*R* ^2^	*F*	*p*
M–indexs	Rare species richness	.005	1.1	.401
	Subdominant species richness	.141	2.6	.014*
	Dominant species richness	.218	8.3	<.001**
	The number of all species	.087	1.6	.105

*M*–*indexes* were treated as dependent variables. The number of the species with different roles was treated as a fixed–effect factor. “Site” (*n* = 4) was treated as random factor to address the nonindependence of quadrats in the same sites. *p <* *.05; **p <* *.001.

## 4 Discussion

### 4.1 Heterogeneous microcommunities under different management modes

Grazing could surpass other environmental factors and become the leading factor controlling plant community (Wang & Li, 1999). Under overgrazing intensity, grazing pressure was evenly spatially distributed over the plant community and all of the populations were suppressed by grazing pressure; only plants such as *Potentilla acaulis, Cleistogenes squarrosa*, and *Carex duriuscula* with low growth form, drought resistance, and strong photosynthetic ability could survive. Therefore, those three plants were the dominant species in the grazing community, and the entire grazing community was almost entirely composed of these three plants (Wang & Li, 1999; Wang, Liang, Liu, & Hao, 2000), leading to a homogeneous community structure and the least heterogeneous microcommunities.

Intermediate disturbance with annual clipping increased biodiversity (Table 1), but the clipping interval, that is, a single growing season, was not long enough for sufficient interspecific competition. Nonselective clipping also resulted in an equal probability and frequency of each species being disturbed, hindering the generation of a dominant species that could win the competition rapidly. After the grazing stopped, some increased plant populations aggregated to form patches during the restoration process in the clipping community. Population patches were an effective organization form for adapting to interspecific competition, and each population partitioned its resources by occupying space (Wang, Liu, He, & Liang, 1996a). The population patches were unstable because one or a few species could not take full advantage of the resources in the space that they occupied; however, the process of species interinfiltration in units of population patches would last longer than species interactions in units of individual plants. Therefore, there were more microcommunity patches in the horizontal structure of the clipping community, and this “metastable state” or “disturbance climax” was expected to last for a long time (Wang, Liu, He, & Liang, 1996b).

In the enclosure community, stratification was apparent; the microcommunities decreased, and many population aggregates began to fuse. Compared to the clipping community, in the enclosure community without interference, more species began to appear in population patches. Because their niche complementarity and interspecific positive interactions then enabled them to better utilize resources, the biomass increased (Cardinale et al., 2012; Hector et al., 1999). High productivity reduced the spatial heterogeneity of the limited resources, making the habitat more homogeneous, and then, interspecific competition became important. The species that had higher resource utilization efficiency (especially for light) thus competitively excluded the subdominant species to become the dominant species (Hautier, Niklaus, & Andy, 2009; Partel, Laanisto, & Zobel, 2007; Rajaniemi, 2002). This competitive exclusion allowed the population patches to gradually fuse, and the physiognomy showed a uniform horizontal structure. The first‐level classification results of TWINSPAN indicated that the main two microcommunities were dominated by *Leymus chinensis* and *Artemisia capillaries* in the enclosure community. *Leymus chinensis* and *Artemisia capillaries* outcompeted the other species and had absolute dominant status, which also confirmed the preliminary fusion of population patches. Thus, the enclosure community had fewer microcommunities than the clipping community.

### 4.2 Soil variables and community structure under different management modes

Grazing, clipping, and enclosure are the three main grassland management modes in Inner Mongolia, and investigation of the change rule of species diversity and ecosystem multifunctionality is beneficial for sustainable grassland management. All species populations were suppressed by grazing pressure in overgrazed grassland, resulting in miniaturization of individual species and narrowing of niche breadth (Wang et al., 2000), and the photosynthetic area decreased (Carrera, Bertiller, & Larreguy, 2008). As a result, the community coverage, height, and productivity all decreased (Chen et al., 2013; Wang et al., 2015), and when the surface vegetation was damaged, the surface barren area and water evapotranspiration both increased (Wang, He, & Zhou, 2002). Additionally, livestock trampling on soil reduced the soil porosity, osmotic force, and water‐holding capacity (Carrera et al., 2008; Wang et al., 2002; Zhang, Han, & Li, 2002). Decreased productivity also reduced litter return, leading to the decline in soil organic matter and nitrogen content (Carrera et al., 2008; Pei, Fu, & Wan, 2008; Wang et al., 2002, 2007). Following the decline in organic matter, soil aggregates and surface crust were damaged (Wang et al., 2010), which resulted in a decrease in clay particles and an increase in sand grains, consequently causing the plant–soil interface to lose balance with concomitant desertification (Wang et al., 2007). The overgrazing community showed all of the above degradation phenomena when compared with the clipping and enclosure communities in this study. Because of the decreased biodiversity and unfavorable soil characteristics, the grazing community had the lowest *M‐index* (Appendix S4). No significant differences were observed in *M‐index* and most soil properties (Table 1) between the clipping and enclosure management modes at the community level, but among heterogeneous microcommunities, the biodiversity, soil variables, and *M‐index* all showed obvious differences.

Therefore, evaluation of *M‐index* at the microcommunity scale might be suitable, whereas quantification of multifunctionality at the community scale might mask the real change rule and relationship between species composition and multifunctionality.

### 4.3 Relationship between biodiversity and multifunctionality

Maestre, Quero, et al. (2012) investigated the relationship between species richness and *M‐index* in 224 ecosystems in arid regions worldwide and found that eight optimal models selected from 255 models all included species richness, indicating that species richness is indispensable for explaining variation in multifunctionality. In existing studies of the relationship between species richness and multifunctionality, because a species played a role not only in one ecosystem function but also in other ecosystem functions, the overlap ratio of species supporting different functions simultaneously was 0.2–0.5 (Hector & Bagchi, 2007). Therefore, with increased ecosystem functions evaluated, species richness was positively saturated with ecosystem functions (Gamfeldt et al., 2008; Hector & Bagchi, 2007). In our study, the number of all species and *M‐index* also showed a significant positive correlation, but subdominant and rare species richness did not have high predictive power for the *M‐index*; only dominant species richness had a better predictive power. Therefore, it is important to distinguish the role and rank of different species in the species richness–multifunctionality model; otherwise, the model might include redundant and unclear information. Thus, species richness would be a poor predictor for multifunctionality if the roles of different species were not distinguished to some degree.

In addition, our identification of a positive correlation between evenness index and *M‐index* was consistent with the results of Maestre, Quero, et al. (2012), who found that species with even distribution could make complementary use of resources more fully, thus increasing ecosystem multifunctionality. Maestre, Castillo‐Monroy, et al. (2012) also found that the interaction of evenness with species richness had a significant effect on *M‐index*, but the relative importance of evenness for multifunctionality was lowest when compared separately with the factor species composition or species richness. These results are not surprising, as the effects of the richness × evenness interaction on ecosystem functioning will largely be driven by individual species. When a dominant species has a strong influence on a given function, a negative relationship between evenness and this particular function would be expected because evenness is inversely proportional to dominance. Therefore, in our study, the *M‐index* of monodominant microcommunities was significantly smaller than that of codominant microcommunities; for example, E1 and E3 were significantly smaller than E2 in the enclosure community. This demonstrates that communities with more dominant species whose distribution is also even might have better multifunctionality.

## 5 Conclusion

1. The different disturbance intensities and intervals of the three management modes led to distinct differences in interspecific and intraspecific relationships and progress of succession; consequently, the overgrazing community had the fewest heterogeneous microcommunities, followed by the enclosure community, whereas the clipping community had the most heterogeneous microcommunities.
2. The soil and vegetation showed apparent degradation in the grazing community, and its *M‐index* was the lowest. There were no significant differences in diversity indices and *M‐index* between the clipping and enclosure communities, but these indicators showed obvious differences among the different microcommunities. Therefore, the microcommunity level would be a more suitable scale to investigate the change rule and relationship between plant species and multifunctionality in seminatural ecosystems.
3. Dominant species richness had stronger explanatory power for ecosystem *M‐index* than subdominant and rare species richness; therefore, it is important to distinguish the role and rank of different species in the species richness–multifunctionality model; otherwise, the model might include inaccurate and redundant information. Communities dominated by more codominant species, in which the codominant species are distributed evenly, might have better ecosystem multifunctionality.

## Conflict of Interest

None declared.
