# Supplementary material for: Heterogeneous microcommunities and ecosystem multifunctionality in seminatural grasslands under three management modes
Source: Ecol Evol. 2016 Nov 27;7(1):14–25. doi: 10.1002/ece3.2604 (PMC5216625; doi:10.1002/ece3.2604)
Supplement: Supplementary file 1 [file ECE3-7-14-s001.docx]

**Appendix S1 General information for 10 microcommunities**

| Microcommunity | Mono-dominance or co-dominance | Layering (Yes or No) | Dominant species | Main companion species |
| --- | --- | --- | --- | --- |
| G1 | Mono-dominance | N | *Cleistogenes squarrosa* | *Potentilla acaulis*，*Carex duriuscula* |
| E1 | Mono-dominance | Y | *Artemisia capillaries* | *Cleistogenes squarrosa*，*Leymus chinensis*, *Serratula centauroides*, *Artemisia frigida*, *Potentilla acaulis* |
| E2 | Co-dominance | Y | *Leymus chinensis*、*Serratula centauroides*、*Artemisia capillaries*, *Caragana microphylla* | *Cleistogenes squarrosa* |
| E3 | Mono-dominance | Y | *Leymus chinensis* | *Caragana microphylla*、*Artemisia capillaries*,  *Serratula centauroides* |
| C1 | Co-dominance | Y | *Artemisia capillaries*, *Cleistogenes squarrosa* | *Leymus chinensis*、*Carex duriuscula*、*Potentilla acaulis*, *Bupleurum tenue* |
| C2 | Mono-dominance | N | *Leymus chinensis* | *Caragana microphylla* |
| C3 | Co-dominance | Y | *Serratula centauroides*, *Artemisia capillaries* | *Cleistogenes squarrosa*, *Potentilla acaulis*, *Carex duriuscula*, *Leymus chinensis*, *Bupleurum tenue*, *Caragana microphylla* |
| C4 | Mono-dominance | N | *Artemisia frigida* | *Potentilla acaulis*, *Cleistogenes squarrosa* |
| C5 | Co-dominance | N | *Poa sphondylodes*, *Cleistogenes squarrosa* | *Bupleurum tenue*、*Serratula centauroides*,  *Artemisia capillaries* |
| C6 | Co-dominance | Y | *Artemisia capillaries,*  *Caragana microphylla* | *Caragana microphylla*、*Artemisia capillaries*,  *Serratula centauroides* |

**Appendix S2 Results of DCA ordination in communities under three management modes.** ○ Grazing quadrats; clipping quadrats; Δ enclosure quadrats.

**Appendix S3 TWINSPAN classification results**

|  |  | Grouping results |
| --- | --- | --- |
| Grazing | Quadrat number | 1 11112 12 11112222  1302958167817404623592345 |
|  | Hierarchical classification result | 0000000000000000000001111  0001111111111111111110011  000000000111111111  001111111000111111 |
| Clipping | Quadrat number | 5 111112221122344 12222123 133344333444545555533455546  623456790137934546014028527892601817934235178160235768948950 |
|  | Hierarchical classification result | 000000000000000000000000000000000000000000011111111111111111  000000000000000000000001111111111111111111100000000011111111  011111111111111111111110000000001111111111100011111100000001  000000000000000111111100000011100000001111 0111110000001 |
| Enclosure | Quadrat number | 6 23334122444433444555455545535­ 2311122122 111233 11235  053579497801233457827890166396572026745501461346121089842938 |
|  | Hierarchical classification results | 000000000000000000000000000000011111111111111111111111111111  000000000000001111111111111111100000000000111111111111111111  000011111111110000000000001111100000000111000000000000001111  011100011111110000000011110001100011111 000000001111110011 |

**Appendix S4** **The differences of biodiversity indices and soil variables between grazing community and clipping community or between grazing community and enclosure community by independent-samples t test.**

|  | Indicator | Grazing community *VS* Clipping community | |  | Grazing community *VS* Enclosure community | |
| --- | --- | --- | --- | --- | --- | --- |
|  |  | t | *p* |  | t | *p* |
| Biodiversity index | Simposon index | -5.57 | < 0.001 |  | -6.78 | < 0.001 |
|  | Marglef index | -6.60 | < 0.001 |  | -6.02 | < 0.001 |
|  | Evenness index | -5.51 | < 0.001 |  | -6.17 | < 0.001 |
| Soil variable | TN | 0.93 | 0.355 |  | -1.54 | 0.127 |
|  | TP | -18.02 | < 0.001 |  | -16.72 | < 0.001 |
|  | AN | -1.64 | 0.105 |  | -3.36 | 0.001 |
|  | AvP | -0.63 | 0.528 |  | 4.09 | < 0.001 |
|  | CEC | -21.88 | < 0.001 |  | -20.33 | < 0.001 |
|  | pH | 0.13 | 0.900 |  | -0.33 | 0.745 |
|  | SMC | -9.64 | < 0.001 |  | -11.28 | < 0.001 |
|  | BD | 17.21 | < 0.001 |  | 15.26 | < 0.001 |
|  | CMC | -9.48 | < 0.001 |  | -10.84 | < 0.001 |
|  | CP | -4.67 | < 0.001 |  | -6.26 | < 0.001 |
|  | NCP | -5.27 | < 0.001 |  | -4.14 | < 0.001 |
|  | SOC | -9.17 | < 0.001 |  | -10.25 | < 0.001 |
| M-index |  | -12.38 | < 0.001 |  | -13.62 | < 0.001 |

**Appendix S5 Species-area curves of communities under three management modes.**
